# Postpartum Hemorrhage-Specific Self-Efficacy Among Obstetric Healthcare Providers in a Tertiary Care Hospital: An Analytical Cross-Sectional Study

**DOI:** 10.3390/healthcare14152406

**Published:** 2026-08-05

**Authors:** Ferhat Aslan, Zehra Çerçer, Sebahat Kuşlu

**Affiliations:** 1 Department of Obstetrics and Gynecology, Faculty of Medicine, Gaziantep Islam Science and Technology University, Gaziantep 27010, Türkiye; 2 Department of Midwifery, Faculty of Health Sciences, Gaziantep Islam Science and Technology University, Gaziantep 27010, Türkiye; cercerzehra@gmail.com; 3 Department of Nursing, Faculty of Health Sciences, Gaziantep Islam Science and Technology University, Gaziantep 27010, Türkiye; sebahatkuslu02@gmail.com

**Keywords:** postpartum hemorrhage, self-efficacy, collective efficacy, healthcare providers, maternal safety, obstetric emergencies

## Abstract

**Highlights:**

**What are the main findings?**

- Obstetric healthcare providers reported high postpartum hemorrhage-specific self-efficacy, with collective efficacy scores exceeding individual self-efficacy scores.
- Individual self-efficacy was significantly associated with professional role, working unit, obstetric experience, PPH case intervention experience, number of PPH cases managed in the previous year, and prior PPH training.

**What are the implications of the main findings?**

- Targeted PPH training programs may consider prioritizing healthcare providers with lower individual self-efficacy and limited obstetric or PPH-related clinical exposure.
- The findings may inform the development of interdisciplinary and simulation-based training strategies targeting self-efficacy in postpartum hemorrhage management.

**Abstract:**

**Background/Objectives**: Postpartum hemorrhage (PPH) remains a major obstetric emergency requiring rapid clinical decision-making, timely intervention, and effective teamwork. Healthcare providers’ self-efficacy is an important factor in perceived confidence and preparedness for managing PPH. This study aimed to determine the PPH-specific individual and collective self-efficacy levels of obstetric healthcare providers working in a tertiary care hospital and to examine associated sociodemographic and professional factors. **Methods**: This analytical and cross-sectional study was conducted among 149 physicians, midwives, and nurses working in obstetrics and gynecology units between 2 and 30 March 2026. Data were collected using a Personal Information Form and the Postpartum Hemorrhage-Specific Self-Efficacy Scale (PHSSES). Descriptive statistics, Mann–Whitney U test, Kruskal–Wallis test, and Spearman correlation analysis were used. **Results**: The mean PHSSES total score was 6.69 ± 0.81, while the mean individual self-efficacy and collective efficacy subscale scores were 6.34 ± 1.00 and 6.90 ± 0.88, respectively. PHSSES total scores differed significantly according to obstetric clinical experience (*p* < 0.05). The self-efficacy subscale was associated with several professional and clinical characteristics, including profession, working unit, obstetric clinical experience, PPH exposure, and previous PPH training (*p* < 0.05). Weekly working hours were positively correlated with PHSSES total and both subscale scores (*p* < 0.05). The most frequently reported PPH interventions were manual uterine massage and uterotonic drug administration. **Conclusions**: Obstetric healthcare providers demonstrated high PPH-specific self-efficacy, with collective efficacy exceeding individual self-efficacy. Individual self-efficacy was associated with professional and clinical characteristics, including PPH experience and previous training. The findings may inform targeted interdisciplinary training strategies for PPH management.

## 1. Introduction

Postpartum hemorrhage (PPH) continues to be a major factor associated with both maternal morbidity and mortality and constitutes a global health problem. It has been reported that approximately 70,000 maternal deaths occur worldwide each year due to PPH [1]. The rapid onset and potential of PPH to become life-threatening within a short period make early diagnosis and timely intervention critical. Therefore, PPH management is a complex process requiring rapid clinical decision-making, correct intervention sequencing, and effective team coordination [2]. In the approach to PPH, not only the knowledge and skill level of the healthcare team, but also their perceptions regarding their own competencies may influence clinical outcomes [3]. Particularly in high-risk situations such as PPH, the self-efficacy perceptions of healthcare workers at both the individual and team levels may directly affect the effectiveness of the intervention process and patient safety [4]. Tertiary care hospitals represent unique clinical environments where healthcare professionals frequently encounter severe PPH cases and are required to make rapid decisions, perform effective interventions, and coordinate teamwork under high-pressure conditions.

The concept of self-efficacy can be defined as a person’s belief in their capacity to apply their knowledge, and it is also shaped by the feelings experienced when successfully performing a task [5]. Obstetric emergencies generally exhibit early warning signs; when these signs are recognized, timely and effective interventions by healthcare workers may be shaped by their self-efficacy perception [6]. Especially in tertiary healthcare institutions, given the intensity of high-risk cases, analyzing healthcare workers’ competency levels regarding obstetric emergencies is important for identifying training needs and developing intervention plans [7,8]. In this context, the self-efficacy level of the healthcare team in postpartum hemorrhage management needs to be investigated to improve maternal safety and clinical outcomes [9].

The literature indicates that studies on PPH management have predominantly been conducted among single professional groups. Existing studies have focused on different samples, including nurses [10,11], midwives [12], physicians [13], or student groups [14,15]. However, few studies have simultaneously evaluated the individual and collective self-efficacy levels of physicians, midwives, and nurses involved together in PPH management within the same clinical setting using the same assessment instrument. Therefore, there is a need for a comprehensive evaluation of self-efficacy levels related to PPH management among different healthcare professional groups.

In addition, a substantial proportion of existing studies have been conducted in different healthcare settings, such as rural hospitals or out-of-hospital healthcare services [4,6,16]. Studies conducted in more advanced healthcare settings have primarily focused on simulation training, teamwork, and clinical performance outcomes; however, studies evaluating healthcare professionals’ individual and collective self-efficacy levels regarding PPH management remain limited [17,18]. Furthermore, studies addressing the concept of collective efficacy have mostly examined it as an outcome variable of simulation-based training programs [3]. Although the Turkish adaptation of the PHSSES, which enables the assessment of both individual and collective self-efficacy, has been conducted [9], no studies evaluating the use of this scale among clinical teams consisting of different healthcare professionals were identified in the available literature.

The primary objective of this study was to determine the postpartum hemorrhage-specific individual and collective self-efficacy levels of obstetric healthcare providers working in a tertiary care hospital. The secondary objective was to examine the associations between self-efficacy levels and sociodemographic and professional characteristics.

## 2. Materials and Methods

### 2.1. Study Design

This was an analytical cross-sectional study. The reporting process for this study complied with the STROBE (Strengthening the Reporting of Observational Studies in Epidemiology) recommendations (Supplementary Materials).

### 2.2. Study Setting and Participants

The study was conducted in the Obstetrics and Gynecology Units of a tertiary care hospital located in a provincial center. The hospital serves as a regional referral center for high-risk pregnancies and obstetric emergencies, provides care to a large population in the surrounding region, and manages approximately 7000 births annually.

The sample size was calculated based on the total score of the Postpartum Hemorrhage-Specific Self-Efficacy Scale (PHSSES), which was the primary outcome of the study. In calculating the sample size, the mean score (6.71) and standard deviation (0.93) reported in the scale’s validity and reliability study were used as references [9]. To determine the sample size capable of detecting a 5% difference from the value in the previous study, an a priori power analysis based on a one-sample mean comparison was conducted using the G*Power 3.1.9.4 (Heinrich Heine University Düsseldorf, Düsseldorf, Germany) soft-ware. The analysis was conducted using a two-tailed test, a significance level of α = 0.05, and 95% power, with an effect size of 0.36. The calculation determined that at least 103 participants were needed for the study to have sufficient power. Finally, the study was completed with 149 healthcare professionals.

Participants aged 18 years and older who had been working as physicians, nurses, or midwives in gynecology and obstetrics units for at least 6 months were included in the study. Those who filled out the questionnaire form incompletely or incorrectly, as well as those who withdrew from the study at any stage, were excluded.

### 2.3. Data Collection and Measurements

Participants were recruited using a non-probability convenience sampling method. All eligible obstetric healthcare providers who were available during the data collection period and agreed to participate were included in the study. Study data were collected between 2 and 30 March 2026 through a questionnaire form created by the researchers, based on self-report. In the hospital setting, questionnaire forms were distributed to participants without collecting personal identification information; participants completed them at their convenience, and the researcher later collected the completed forms. All questionnaires were completed anonymously.

The study utilized a Personal Information Form and the Postpartum Hemorrhage-Specific Self-Efficacy Scale (PHSSES).

Personal Information Form: This form was prepared by the researchers and consisted of 10 questions inquiring about the sociodemographic and clinical experience characteristics of participants. The collected variables included age, gender, profession, weekly working hours, working unit, total years of work experience, clinical experience in obstetrics, previous PPH training status, history of intervention in PPH cases, and the number of PPH cases managed within the previous year. Participants who reported previous experience in managing PPH cases were asked to indicate the types of interventions they had performed throughout their professional careers. Multiple responses were allowed, as participants could report more than one intervention.

Postpartum Hemorrhage-Specific Self-Efficacy Scale: This scale, adapted by Coşkuner Potur et al. (2021) [9], evaluates both individual and team self-efficacy in PPH management. Items 1–8 assess individual efficacy, and items 9–21 assess collective efficacy. Responses are scored from 1 (Never) to 8 (Always), and all items except item 2 are reverse-scored. The scale score is calculated as the mean item score, with scores ranging from 1 to 8. Higher scores indicate higher levels of PPH-specific self-efficacy, whereas lower scores indicate lower self-efficacy. The scale has high reliability, with Cronbach’s alpha values of 0.92 for the overall scale, 0.91 for the individual self-efficacy subscale, and 0.97 for the collective efficacy subscale [9]. In the present study, Cronbach’s alpha values were 0.941 for the total scale, 0.866 for the self-efficacy subscale, and 0.962 for the collective efficacy subscale.

### 2.4. Ethical Aspects

The study received ethical clearance from the Gaziantep City Hospital Non- Interventional Clinical Research Ethics Committee (Approval no: 456/2026; Date of approval: 18 February 2026). Additionally, the necessary official permission was obtained from the hospital where the research was conducted. At the outset of the study, participants were informed about the aims and scope of the research, as well as their right to withdraw at any stage, and written informed consent was obtained. Permission to use the scale was secured via email from the authors who established its validity and reliability. The study was carried out in line with the ethical principles outlined in the Declaration of Helsinki.

### 2.5. Data Analysis

Data were analyzed using SPSS version 27 (IBM Corp., Armonk, NY, USA). Normality was checked with the Kolmogorov–Smirnov test and histograms, indicating a non-normal distribution. Descriptive statistics included frequencies, percentages, means, and standard deviations. Comparisons between two groups were performed with the Mann–Whitney U test, while the Kruskal–Wallis test was used for multiple groups. Spearman correlation assessed the relationship between weekly working hours and scale scores. A *p*-value of less than 0.05 was considered statistically significant.

## 3. Results

A total of 161 healthcare professionals were employed in the clinics included in the study. Two professionals did not meet the inclusion criteria and were excluded. Two professionals were excluded due to incomplete questionnaire responses. Of the remaining 157 eligible professionals, five declined to participate, and three were on leave during the data collection period. Therefore, the study was completed with 149 participants, resulting in a response rate of 94.9%.

In the study, the mean age of participants was 27.5 years and the mean weekly working time was 52.4 h; the majority were female (87.9%) and midwives (54.4%). When working durations were examined, 42.3% had 2 years or less of total working experience, and 41.6% had between 3 and 10 years of total working experience. In addition, 53.0% had 2 years or less of obstetric experience. Overall, 91.9% of participants had intervened in at least one PPH case throughout their professional lives, 43.6% had intervened in 1–5 PPH cases in the last year, and 69.8% had previously received PPH training (Table 1).

Participants’ PHSSES total score was 6.69 ± 0.81, the individual self-efficacy subscale mean was 6.34 ± 1.00, and the collective self-efficacy subscale mean was 6.90 ± 0.88 (Table 2).

The comparison of PHSSES total and subscale scores according to the personal characteristics of healthcare professionals is presented in Table 3. PHSSES total scores differed significantly only according to clinical experience in obstetrics (*p* = 0.038); however, Bonferroni-adjusted pairwise comparisons revealed no significant differences between the groups (*p* > 0.05). Self-efficacy subscale scores varied significantly by gender, profession, working unit, total years of work, clinical experience in obstetrics, PPH case intervention status, the number of PPH cases managed during the previous 12 months, and previous PPH training status (Table 3).

A significant difference was found in self-efficacy scores according to profession (*p* = 0.001). Bonferroni-adjusted post hoc comparisons showed that nurses had significantly lower self-efficacy scores than midwives (adjusted *p* = 0.035) and physicians (adjusted *p* = 0.001), whereas no significant difference was observed between midwives and physicians. Self-efficacy scores also differed significantly according to working unit (*p* = 0.002). Post hoc analysis demonstrated that professionals working in all obstetrics and gynecology units had significantly higher self-efficacy scores compared with those working in obstetrics and gynecology wards (adjusted *p* = 0.001). Regarding total years of work, a significant difference was found in self-efficacy scores (*p* = 0.011). Professionals with ≥10 years of work experience had significantly higher self-efficacy scores than those with ≤2 years of experience (adjusted *p* = 0.016) (Table 3).

Self-efficacy scores also differed significantly according to clinical experience in obstetrics (*p* < 0.001). Bonferroni-adjusted pairwise comparisons showed that professionals with ≤2 years of obstetric clinical experience had significantly lower self-efficacy scores compared with those with 3–10 years (adjusted *p* = 0.004) and ≥10 years of experience (adjusted *p* = 0.001). A significant difference was found in self-efficacy scores according to the number of PPH cases managed during the previous 12 months (*p* = 0.014). Professionals who had managed ≥10 PPH cases had significantly higher self-efficacy scores than those who had not managed any cases (adjusted *p* = 0.002) and those who had managed 1–5 cases (adjusted *p* = 0.022). No significant differences were observed in collective efficacy scores according to personal characteristics (*p* > 0.05). Positive correlations were found between weekly working hours and PHSSES total, self-efficacy, and collective efficacy scores (r = 0.269, *p* < 0.001; r = 0.268, *p* < 0.001; and r = 0.203, *p* = 0.013, respectively) (Table 3).

The most frequently reported interventions for PPH management were manual uterine massage (93.4%) and uterotonic administration (92.7%). Blood transfusion (78.1%), Bakri balloon/tamponade (56.9%), and surgical interventions (29.2%) were reported less frequently (Table 4).

## 4. Discussion

In this study, participants were found to have high PPH-specific self-efficacy scores. The higher collective efficacy scores suggest that healthcare professionals perceive greater confidence in their team’s ability to manage PPH-related situations compared with their perceptions of their own individual capabilities. It is known that a multidisciplinary team must conduct obstetric emergency care and that effective teamwork strengthens clinical decision-making and intervention processes [19]. For this reason, healthcare professionals may have perceived higher collective efficacy, which may reflect the role of team support and coordination in obstetric emergency care.

Collective efficacy scores among healthcare professionals did not differ significantly according to any personal characteristic variable. This finding suggests that, as a team-based construct, collective efficacy may be shaped more by team dynamics and shared clinical practices rather than by individual characteristics.

The present study found positive associations between weekly working hours and both individual and collective self-efficacy scores. This finding may be explained by the increased exposure to obstetric situations and greater opportunities for clinical practice among healthcare professionals with longer working hours. Consistent with this interpretation, previous research has reported that healthcare professionals with greater clinical exposure demonstrate higher levels of self-efficacy in managing obstetric emergencies [13]. Regarding gender, a significant difference was observed only in the self-efficacy subscale, with female professionals reporting lower scores than male professionals. Similarly, a study among obstetrics and gynecology physicians found that female physicians reported lower confidence in practical and clinical skills compared with male physicians, suggesting possible gender-related differences in perceived professional confidence [20]. These findings suggest that differences in perceived self-efficacy among healthcare professionals may reflect variations in confidence rather than differences in actual clinical competence, highlighting the need for supportive learning environments that promote confidence development.

A significant difference in the self-efficacy subscale was observed according to professional group. Post hoc comparisons showed that nurses had lower self-efficacy scores than both physicians and midwives, whereas no significant difference was found between physicians and midwives. This finding may partly be related to differences in educational background, clinical exposure, and professional roles among healthcare professionals. Physicians receive extensive medical training, midwives receive specialized obstetric education, whereas nurses typically receive obstetrics and gynecology education as part of their broader nursing curriculum. Previous research has indicated that longer and more intensive educational experiences may contribute to higher self-efficacy levels [21]. In a study conducted in Iran, midwives had higher clinical performance self-efficacy compared with other healthcare workers; therefore, managers and policymakers involved in health promotion and planning processes should make plans accordingly [22]. Therefore, identifying areas where nurses may require additional support regarding PPH management and strengthening undergraduate and in-service training programs may contribute to improving self-efficacy. However, these differences should not be interpreted as indicators of professional competence, but rather as reflections of variations in educational exposure, clinical experience, and professional responsibilities.

In the study, significant differences were found between total working years, obstetric clinical experience, PPH case intervention status, and number of interventions and the individual self-efficacy subscale; healthcare workers with more experience reported higher self-efficacy perception. This finding is consistent with self-efficacy theory. Completing a task strengthens an individual’s perception of competence and confidence regarding that task [23]. Therefore, increased clinical exposure and greater experience in managing PPH cases may be associated with higher self-efficacy among healthcare professionals; however, these findings should be interpreted cautiously due to differences in subgroup sizes. In addition, participants who had previously received PPH training had significantly higher self-efficacy scores. Previous studies have demonstrated that theoretical, practical, and simulation-based training programs can positively influence healthcare professionals’ knowledge, clinical performance, and self-efficacy regarding postpartum hemorrhage management [10,14,24].

A significant difference was found in self-efficacy scores according to working unit. Professionals working across multiple obstetrics and gynecology units had higher self-efficacy scores compared with those working only in obstetrics and gynecology wards. This finding may be associated with differences in the level of clinical exposure among healthcare professionals. According to self-efficacy theory, direct and successful performance experiences are important sources that shape perceived competence [5]. Therefore, working in different clinical units and having opportunities to encounter various clinical situations may support the development of healthcare professionals’ self-efficacy regarding PPH management.

In our study, participants most frequently intervened in PPH cases using manual uterine massage and uterotonic drug administration. In contrast, blood transfusion, Bakri balloon tamponade, and surgical interventions were applied less frequently. This pattern is consistent with the stepwise approach recommended in clinical guidelines, in which medical and minimally invasive interventions are generally considered first-line approaches, while surgical procedures are reserved for cases that do not respond adequately to initial management strategies [2,25]. The tertiary hospital setting of the study may explain the frequency and variety of PPH interventions reported by healthcare professionals, as referral centers often manage more severe and complex PPH cases. Furthermore, greater exposure to diverse PPH management procedures may be associated with higher clinical confidence and self-efficacy among healthcare professionals.

### Strengths and Limitations

This study provides original and clinically valuable data by encompassing physicians, midwives, and nurses in a tertiary care hospital and measuring individual and collective self-efficacy in PPH management with a validated and reliable scale. However, several limitations should be considered. The study was conducted in a single center, which may limit the generalizability of the findings. The unequal distribution of participants across professional groups and the relatively low number of male participants may have limited the interpretation of subgroup comparisons. Additionally, the voluntary participation of healthcare professionals may have introduced selection bias. Data regarding previous PPH experiences and interventions were based on participants’ self-reports, which may be subject to recall bias. Furthermore, the cross-sectional design of the study does not allow causal relationships to be established. Although the potential influence and interrelationships of personal and professional characteristics were considered, multivariable analysis was not performed due to the study objective and sample size constraints, which limits the ability to determine the independent associations of factors related to self-efficacy. The study also did not assess participants’ actual clinical performance; therefore, self-efficacy perceptions may not necessarily reflect objective competence in PPH management.

## 5. Conclusions

Obstetric healthcare providers demonstrated high levels of self-efficacy in postpartum hemorrhage management. Higher individual self-efficacy scores were observed among professionals with previous PPH training, greater obstetric clinical experience, and more frequent exposure to PPH cases. Uterotonic agents and uterine massage were the most commonly reported interventions, whereas invasive interventions were less frequently performed. These findings highlight the importance of maintaining effective education and clinical exposure opportunities to support healthcare professionals’ preparedness for PPH management. Targeted interdisciplinary and simulation-based training strategies may be considered as potential approaches to support the development of individual and collective self-efficacy in postpartum hemorrhage management. Further studies using longitudinal designs are needed to evaluate changes in self-efficacy over time and determine the effects of training interventions.

## Figures and Tables

**Table 1:** Distribution of healthcare professionals’ personal characteristics (n = 149).

Personal Characteristics	n	%
Age, years, mean ± SD (min–max) = 27.05 ± 5.39 (22–49)		
Weekly working hours, mean ± SD (min–max) = 52.45 ± 13.21 (40–90)		
Gender		
Female	131	87.9
Male	18	12.1
Profession		
Physician	41	27.5
Midwife	81	54.4
Nurse	27	18.1
Working unit		
Labor and Delivery Unit	37	24.8
Obstetrics and Gynecology Wards	73	49.0
All Obstetrics and Gynecology Units *	39	26.2
Total years of work ^#^		
≤2 years	63	42.3
3–10 years	62	41.6
≥10 years	24	16.1
Clinical experience in obstetrics		
≤2 years	79	53.0
3–10 years	52	34.9
≥10 years	18	12.1
PPH case intervention		
Yes	137	91.9
No	12	8.1
Number of PPH cases intervened in the last year.		
None in the last 12 months	24	16.1
1–5	65	43.6
5–10	28	18.8
≥10 cases	32	21.5
Previous PPH training status		
Received	104	69.8
Not received	45	30.2

* Physicians rotate through all units. ^#^ The maximum number of years of work was 29. PPH: postpartum hemorrhage; SD: standard deviation.

**Table 2:** Mean total and subscale scores of the Postpartum Hemorrhage-Specific Self-Efficacy Scale (PHSSES) among participants.

Scale	Mean ± Standard Deviation	Min–Max
PHSSES total	6.69 ± 0.81	3–8
Self-efficacy subscale	6.34 ± 1.00	3–8
Collective efficacy subscale	6.90 ± 0.88	3–8

**Table 3:** Comparison of personal characteristics and Postpartum Hemorrhage-Specific Self-Efficacy Scale (PHSSES) total and subscale scores of healthcare professionals (n = 149).

Personal Characteristics	PHSSES Total Mean ± SD	Test and *p*-Value	Self-Efficacy Mean ± SD	Test and *p*-Value	Collective Efficacy Mean ± SD	Test and *p*-Value
Gender						
Female	6.65 ± 0.83	MWU = 977.00 *p* = 0.239	6.26 ± 0.99	MWU = 677.00 ***p* = 0.003**	6.90 ± 0.91	MWU = 1134.00 *p* = 0.793
Male	6.93 ± 0.62		6.95 ± 0.89		6.92 ± 0.62	
Profession						
Physician	6.91 ± 0.63	KW = 4.037 *p* = 0.133	6.69 ± 0.87 ^a^	KW = 13.118 ***p* = 0.001**	7.05 ± 0.70	KW = 1.014 *p* = 0.239
Midwife	6.68 ± 0.74		6.37 ± 0.92 ^a^		6.87 ± 0.82	
Nurse	6.36 ± 1.12		5.71 ± 1.15 ^b^		6.76 ± 1.24	
Working unit						
Labor and Delivery Unit	6.71 ± 0.55	KW = 5.954 *p* = 0.051	6.43 ± 0.89 ^ab^	KW = 12.262 ***p* = 0.002**	6.89 ± 0.65	KW = 2.135 *p* = 0.344
Obstetrics and Gynecology Wards	6.51 ± 0.97		6.05 ± 1.08 ^a^		6.80 ± 1.06	
All Obstetrics and Gynecology Units *	6.99 ± 0.57		6.80 ± 0.74 ^b^		7.11 ± 0.66	
Total years of work ^#^						
≤2 years	6.56 ± 0.83	KW = 2.879 *p* = 0.237	6.08 ± 1.01 ^a^	KW = 9.032 ***p* = 0.011**	6.85 ± 0.87	KW = 0.951 *p* = 0.621
3–10 years	6.77 ± 0.86		6.44 ± 1.03 ^ab^		6.97 ± 0.93	
≥10 years	6.82 ± 0.61		6.77 ± 0.69 ^b^		6.86 ± 0.78	
Clinical experience in obstetrics						
≤2 years	6.52 ± 0.88	KW = 6.539 ***p* = 0.038**	6.03 ± 1.04 ^a^	KW = 17.898 ***p* < 0.001**	6.82 ± 0.96	KW = 0.898 *p* = 0.638
3–10 years	6.87 ± 0.71		6.61 ± 0.87 ^b^		7.02 ± 0.79	
≥10 years	6.93 ± 0.58		6.94 ± 0.63 ^b^		6.92 ± 0.79	
PPH case intervention						
Yes	6.73 ± 0.80	MWU = 559.00 *p* = 0.066	6.41 ± 0.97	MWU = 404.00 ***p* = 0.004**	6.92 ± 0.87	MWU = 738.00 *p* = 0.557
No	6.26 ± 0.87		5.50 ± 1.01		6.73 ± 1.06	
Number of PPH cases intervened in the last year.						
None in the last 12 months	6.30 ± 0.85	KW = 1.184 *p* = 0.553	5.81 ± 1.08 ^a^	KW = 8.477 ***p* = 0.014**	6.60 ± 0.93	KW = 0.088 *p* = 0.957
1–5	6.66 ± 0.88		6.21 ± 1.05 ^a^		6.93 ± 0.99	
5–10	6.84 ± 0.65		6.52 ± 0.81 ^ab^		7.03 ± 0.68	
≥10 cases	6.91 ± 0.66		6.84 ± 0.74 ^b^		6.96 ± 0.74	
Previous PPH training status						
Received	6.77 ± 0.67	MWU = 2080.00 *p* = 0.282	6.50 ± 0.85	MWU = 1795.00 ***p* = 0.024**	6.94 ± 0.78	MWU = 2331.50 *p* = 0.972
Not received	6.50 ± 1.05		5.98 ± 1.21		6.81 ± 1.09	
Weekly working hours	PHSSES total r and *p*-value		Self-efficacy r and *p*-value		Collective efficacy r and *p*-value	
	r = 0.269 ***p* < 0.001**		r = 0.268 ***p* < 0.001**		r = 0.203 ***p* = 0.013**	

* Physicians rotate through all units. ^#^ The maximum number of years of work was 29. PPH: postpartum hemorrhage; PHSSES: Postpartum Hemorrhage-Specific Self-Efficacy Scale; SD: standard deviation; MWU: Mann–Whitney U test; KW: Kruskal–Wallis test; r: Spearman correlation; Different superscript letters indicate significant differences according to Bonferroni-adjusted pairwise comparisons. Values shown in bold indicate statistically significant differences (*p* < 0.05).

**Table 4:** Types of interventions performed by participants during postpartum hemorrhage management throughout their professional careers (n = 137).

Type of Intervention	n	%
Uterotonic drug administration	127	92.7
Manual uterine massage	128	93.4
Blood transfusion	107	78.1
Surgical intervention	40	29.2
Bakri balloon/tamponade	78	56.9

Participants were asked to report the types of interventions they had performed during PPH management throughout their professional careers. Some participants reported more than one type of intervention.

## Data Availability

The data presented in this study are available from the corresponding author upon reasonable request. The data are not publicly available due to privacy and ethical restrictions.

## Supplementary Materials

The following supporting information can be downloaded at https://www.mdpi.com/article/10.3390/healthcare14152406/s1, The STROBE checklist is available as a Supplementary File.

## Abbreviations

The following abbreviations are used in this manuscript:

PPH	Postpartum hemorrhage
PHSSES	Postpartum Hemorrhage-Specific Self-Efficacy Scale
SD	Standard deviation
MWU	Mann–Whitney U test
KW	Kruskal–Wallis test
SPSS	Statistical Package for the Social Sciences
STROBE	Strengthening the Reporting of Observational Studies in Epidemiology
